# Traite Elementaire d’Anatomie Medical du Systeme Nerveux

**Published:** 1886-10

**Authors:** 


Traite Elementaire d'Anatomie Medical du Systeme Nerveux. *Par* Ch. Fere, *Médecin-adjoint de la Sapêltrière, Paris. Aux Bureaux du Progrés Médical. Octavo, pp. 493. 1886.*

The reader expecting to find in this work anything original, or valuable discussions on the mooted points in cerebral anatomy, will be greatly disappointed. Yet the work is of value and should be translated into English, as we have no similar work in our tongue. The merit of the work lies in its clear and concise arrangement, and the successful application of anatomical facts to the elucidation of diagnostic problems,—the grand difficulty for the beginner in neurology.

